# Improved Confidence Intervals for the Youden Index

**DOI:** 10.1371/journal.pone.0127272

**Published:** 2015-07-01

**Authors:** Guogen Shan

**Affiliations:** Epidemiology and Biostatistics Program, Department of Environmental and Occupational Health School of Community Health Sciences, University of Nevada Las Vegas, Las Vegas, NV 89154, USA; University of East Piedmont, ITALY

## Abstract

The Youden Index is a summary measurement of the receiver operating characteristic (ROC) curve for the accuracy of a diagnostic test with ordinal or continuous endpoints. The bootstrap confidence interval based on the adjusted proportion estimate was shown to have satisfactory performance among the existing confidence intervals, including the parametric interval via the delta method. In this article, we propose two confidence intervals using the square-and-add limits based on the Wilson score method. We compare the two proposed intervals with the existing interval with extensive simulation studies. The new interval based on the empirical proportion estimate generally has better performance than that based on the adjusted proportion estimate. A real example from a clinical trial of prostate cancer is illustrated for the application of the new intervals.

## Introduction

For a diagnostic test with ordinal or continuous endpoints, a receiver operating characteristic (ROC) curve has been widely used to measure the accuracy of the diagnostic test [1, 2]. The ROC curve is created by plotting the sensitivity vs 1 minus specificity for various cut points, and the x-axis of the ROC is 1–specificity. The cut point is used to determine the diagnostic results, e.g., positive or negative, diseased or healthy. The range of the cut point is generally from −∞ to +∞. It is of interest to find the optimal cut point to increase the accuracy of a diagnostic test [3].

The Youden Index (J) [4] is a well known measurement for the ROC curve to measure the clinical diagnostic ability of a test. It is defined as

$$\begin{array}{c} J=\max_{c}[Sen(c)+Spe(c)-1], \end{array}$$

where *c* is the cut point. Diagnostic tests with higher *J* values would be preferable. The Youden Index is an optimal trade-off between sensitivity and specificity with an equal weight being assigned to sensitivity and specificity. For given total sample sizes in the diseased group and the non-diseased group, the optimal cut point would lead the maximum number of subjects being correctly diagnosed. Although the theoretical range of the Youden Index is from -1 to 1, the practical range in use is often from 0 to 1 since negative values of the Youden Index do not have meaningful interpretation in practice. *J* = 1 represents a prefect diagnostic test and *J* = 0 indicates that the diagnostic test is not effective to determine the disease status. The Youden Index has been applied in many statistical and medical applications [3, 1].

Fluss et al. [5] were among the first to introduce nonparametric confidence intervals for the Youden Index. Specifically, the empirical distributions are used to estimate the Youden Index and its associated confidence interval. The coverage probability of the bootstrap based confidence intervals is generally less than the nominal level [3]. Later, Schisterman and Perkins [3] used the delta method [6] to improve the coverage probability of the confidence interval when the underlying distributions are normal or gamma. This parametric approach works well as compared to three bootstrap confidence intervals: the bootstrap percentile interval, the bias corrected and accelerated interval, and the asymptotic bootstrap interval based on bootstrap mean and variance. Very recently, Zhou and Qin [7] proposed two bootstrap intervals based on the adjusted estimate for a binomial proportion by Agresti and Coull [8] (referred to as the AC estimate). They showed that the bootstrap confidence intervals are comparable to the parametric interval via the delta method when the underlying distributions are correctly specified, and have better performance when the the distributions are misspecified. Among the two proposed intervals, the one based on the bootstrap mean and variance has better performance than the bootstrap percentile interval.

The variance of the estimated Youdex Index is estimated by parametric or nonparametric approaches in the existing methods for the confidence interval of the Youden Index. An alternative would be that one treats the parameter in the variance as an unknown quantity, and the confidence interval is then obtained by solving an equality. This method, called the variance profile method, is also known as the Wilson score method [9, 10, 11, 12]. In this article, we utilize the Wilson score method to construct the confidence interval of the Youden Index. The Youden Index can be rewritten as the difference between two independent proportions when the optimal cut point is determined. For each proportion, the Wilson score method will be used to compute the confidence interval of the proportion. The confidence interval of the Youden Index is then constructed by the square-and-add method [13]. The binomial proportion can be estimated by the empirical estimate or the adjusted estimate for the proportion. Therefore, we propose two new confidence intervals using the square-and-add limits based on the Wilson score method. Extensive Monte Carlo simulation studies are conducted for comparing the proposed intervals with the existing intervals.

The rest of this article is organized as follows. In Section 2, we briefly review the bootstrap confidence interval based on the adjusted proportion estimate (the AC estimate), and propose two new confidence intervals using the square-and-add limits based on the Wilson score method. We then conduct Monte Carlo simulation studies to compare the new and existing confidence intervals with regard to coverage probability and width in Section 3. An example from a clinical study on prostate cancer is illustrated to show the usage of the proposed confidence intervals at the end of Section 3. Section 4 is given to remarks.

## Confidence intervals

Suppose *X* and *Y* are diagnostic results for the patients from the non-diseased group and the diseased group, respectively. It is reasonable to assume the independence between *X* and *Y*. For a given cut point *c*, sensitivity and specificity of a diagnostic test are defined as

$$\begin{array}{c} Sen(c)=P(Y\geq c)\;\text{and}\;Spe(c)=P(X\leq c). \end{array}$$

The Youden Index [4] is expressed as

$$\begin{array}{c} J=\max_{c}[Sen(c)+Spe(c)-1]=\max_{c}[P(X\leq c)-P(Y<c)]. \end{array}$$

The *J* is a measurement to assess the sensitivity and specificity simultaneously, and it is obtained by plugging in the optimal cut point c* such that it maximizes the quantity *P*(*X* ≤ *c*)−*P*(*Y* < *c*).

Let *X*_1_, *X*_2_, ⋯, *X*_*m*_, and *Y*_1_, *Y*_2_, ⋯, *Y*_*n*_ be the observations from the non-diseased group and diseased group, respectively. The Youden Index can be estimated as

$$\begin{array}{c} \hat{J}=\max_{c}[\frac{\sum_{i=1}^{m}I\left(X_{i}\leq c\right)}{m}-\frac{\sum_{j=1}^{n}I\left(Y_{j}<c\right)}{n}] \end{array}$$ (1)

where *I*(*D*) is an indicator function, with *I*(*D*) = 1 if *D* is true, 0 otherwise. In this article, we focus on the construction of two-sided confidence intervals for the Youdex Index *J*.

### 2.1 Bootstrap confidence interval

Zhou and Qin [7] proposed a bootstrap confidence interval for the Youden Index based on the AC estimate [8] for a binomial proportion. The Youden Index is estimated as

$$\begin{array}{c} {\hat{J}}_{AC}=\max_{c}[\frac{\sum_{i=1}^{m}I\left(X_{i}\leq c\right)+\frac{1}{2}z_{1-\alpha /2}^{2}}{m+z_{1-\alpha /2}^{2}}-\frac{\sum_{j=1}^{n}I\left(Y_{j}<c\right)+\frac{1}{2}z_{1-\alpha /2}^{2}}{n+z_{1-\alpha /2}^{2}}], \end{array}$$ (2)

where *z*_1−*α*/2_ is the 1−*α*/2 percentile of a standard normal distribution. The quantities $\frac{1}{2}z_{1-\alpha /2}^{2}$ and $z_{1-\alpha /2}^{2}$ are added in the numerator and denominator as compared to the estimate of *J* in Eq (1). When *α* = 0.05, $z_{1-\alpha /2}^{2}$ is close to 4. This may be viewed as adding two successes and two failures in the study.

We denote the samples for the non-diseased group and the diseased group with **x** = (*x*_1_, *x*_2_, ⋯, *x*_*m*_) and **y** = (*y*_1_, *y*_2_, ⋯, *y*_*n*_), respectively. The bootstrap samples from each group are obtained to calculate the Youden Index estimate, ${\hat{J}}_{AC}$. Let the bootstrap samples be x* and y*, where x* are *m* samples from **x** and y* are *n* samples from **y** with replacement. The Youden Index estimate, ${\hat{J}}_{AC}^{*}$, can be computed from the Eq (2) using the bootstrap samples x* and y*. This resampling procedure is repeated B times to generate B Youden Index estimates, ${\hat{J}}_{{AC}_{1}}^{*},{\hat{J}}_{{AC}_{2}}^{*},\cdots ,{\hat{J}}_{{AC}_{B}}^{*}$. The bootstrap mean and variance estimates of the Youden Index are calculated as

$$\begin{array}{c} \bar{{\hat{J}}_{AC}^{*}}=\frac{\sum_{k=1}^{B}{\hat{J}}_{{AC}_{k}}^{*}}{B}, \end{array}$$

$$\begin{array}{c} \hat{Var}\left({\hat{J}}_{AC}^{*}\right)=\frac{1}{B-1}\sum_{k=1}^{B}{\left({\hat{J}}_{{AC}_{k}}^{*}-\bar{{\hat{J}}_{AC}^{*}}\right)}^{2}. \end{array}$$

The corresponding Bootstrap confidence interval using the AC estimate (referred to as the BAC confidence interval) is

$$\begin{array}{c} (\bar{{\hat{J}}_{AC}^{*}}-z_{1-\alpha /2}\sqrt{\hat{Var}\left({\hat{J}}_{AC}^{*}\right)}\;,\;\bar{{\hat{J}}_{AC}^{*}}+z_{1-\alpha /2}\sqrt{\hat{Var}\left({\hat{J}}_{AC}^{*}\right)}). \end{array}$$ (3)

The BAC confidence interval is based on the AC estimate for the Youden Index. This confidence interval can be obtained by invoking the central limit theorem. The Youden Index can also be estimated for Eq (1) with an empirical estimate. The confidence interval construction would be similar to that based on the AC estimate, and the only difference is the estimate of the Youden Index from each resampling step. This confidence interval based on $\hat{J}$ was studied by Schisterman and Perkins [3]. They showed that this bootstrap confidence interval is not as good as the intervals based on parametric approaches with regard to the coverage probability and width.

Later, Zhou and Qin [7] compared the bootstrap confidence interval based on the AC estimate for the Youden Index with the parametric confidence interval via the delta method [3]. In addition to the bootstrap confidence interval based on the AC estimate, Zhou and Qin [7] also considered the percentile bootstrap confidence interval based on the AC estimate for the Youden Index. They concluded the two bootstrap confidence intervals based on the AC estimate for the Youden Index are comparable to the parametric intervals when the distribution assumptions are met, and outperform the parametric intervals when the distributions are misspecified. The BAC interval is generally better than the percentile bootstrap interval. For this reason, the BAC interval is chosen for comparison in this article.

### 2.2 Two new confidence intervals

The existing confidence intervals are Wald-type confidence intervals. The variance is estimated by different methods, such as the delta method and the bootstrap method. Based on the existing literature, the coverage property is generally not satisfactory. In addition, only medium to large sample sizes are considered in existing literature.

We consider the square-and-add limits based on the Wilson score method [9], to construct the confidence interval for the Youden Index. When the optimal cut point, c*, is determined, the Youden Index, *J*, can be expressed as the difference between two independent proportions

$$\begin{array}{c} J=P\left(X\leq c^{*}\right)-P\left(Y<c^{*}\right). \end{array}$$

For simplicity, let *p*_1_ = *P*(*X* ≤ c*) and *p*_2_ = *P*(*Y* < c*). The Wilson confidence intervals for *p*_1_, (*l*_1_, *u*_1_), are the roots of the following equality

$$\begin{array}{c} {\left(p_{1}-{\hat{p}}_{1}\right)}^{2}=z_{1-\alpha /2}^{2}\frac{p_{1}\left(1-p_{1}\right)}{m}. \end{array}$$

It is easy to show that

$$\begin{array}{c} l_{1}=\frac{1}{1+z_{1-\alpha /2}^{2}/m}[{\hat{p}}_{1}+\frac{z_{1-\alpha /2}^{2}}{2m}-z_{1-\alpha /2}\sqrt{\frac{{\hat{p}}_{1}\left(1-{\hat{p}}_{1}\right)}{m}+\frac{z_{1-\alpha /2}^{2}}{4m^{2}}}\;], \end{array}$$

and

$$\begin{array}{c} u_{1}=\frac{1}{1+z_{1-\alpha /2}^{2}/m}[{\hat{p}}_{1}+\frac{z_{1-\alpha /2}^{2}}{2m}+z_{1-\alpha /2}\sqrt{\frac{{\hat{p}}_{1}\left(1-{\hat{p}}_{1}\right)}{m}+\frac{z_{1-\alpha /2}^{2}}{4m^{2}}}\;]. \end{array}$$

Similarly, (*l*_2_, *u*_2_), the Wilson confidence intervals for *p*_2_, are the roots of

$$\begin{array}{c} {\left(p_{2}-{\hat{p}}_{2}\right)}^{2}=z_{1-\alpha /2}^{2}\frac{p_{2}\left(1-p_{2}\right)}{n}. \end{array}$$

It follows that

$$\begin{array}{c} l_{2}=\frac{1}{1+z_{1-\alpha /2}^{2}/n}[{\hat{p}}_{2}+\frac{z_{1-\alpha /2}^{2}}{2n}-z_{1-\alpha /2}\sqrt{\frac{{\hat{p}}_{2}\left(1-{\hat{p}}_{2}\right)}{n}+\frac{z_{1-\alpha /2}^{2}}{4n^{2}}}\;], \end{array}$$

and

$$\begin{array}{c} u_{2}=\frac{1}{1+z_{1-\alpha /2}^{2}/n}[{\hat{p}}_{2}+\frac{z_{1-\alpha /2}^{2}}{2n}+z_{1-\alpha /2}\sqrt{\frac{{\hat{p}}_{2}\left(1-{\hat{p}}_{2}\right)}{n}+\frac{z_{1-\alpha /2}^{2}}{4n^{2}}}\;]. \end{array}$$

The confidence interval of *J* is calculated as [13]

$$\begin{array}{c} \left(J_{L},J_{U}\right)=\left(\hat{J}-A,\hat{J}+B\right), \end{array}$$

where

$$\begin{array}{c} A=z_{1-\alpha /2}\sqrt{\frac{l_{1}\left(1-l_{1}\right)}{m}+\frac{u_{2}\left(1-u_{2}\right)}{n}}, \end{array}$$

$$\begin{array}{c} B=z_{1-\alpha /2}\sqrt{\frac{u_{1}\left(1-u_{1}\right)}{m}+\frac{l_{2}\left(1-l_{2}\right)}{n}}. \end{array}$$

The estimates ${\hat{p}}_{1}$ and ${\hat{p}}_{2}$ can be obtained by Eqs (1) and (2). Different estimates of ${\hat{p}}_{1}$ and ${\hat{p}}_{2}$ would lead to different confidence intervals for *p*_1_ and *p*_2_, and further affect the final confidence interval estimates of the Youden Index. We refer to the confidence intervals using Eq (1) as the NP method, and Eq (2) as the NPAC method.

Unlike the Wald-type confidence interval, the parameter in the variance is considered as an unknown parameter in the Wilson score method. The Wilson confidence intervals are then obtained by finding the roots of two equations. This method may be able to improve the coverage probability of the confidence interval [14, 15]. This method has been successfully applied in many important statistical research areas [15, 11].

## Simulation study

We compare the performance of the bootstrap BAC interval, the NP interval, and the NPAC interval with regards to the coverage probability and width by using extensive Monte Carlo simulations. The nominal level of coverage is set as 95% (*α* = 0.05). Sixteen sample size combinations are considered: (*m*, *n*) = (20, 20), (20, 40), (20, 60), (40, 20), (40, 40), (40, 80), (60, 30), (60, 60), (60, 90), (80, 60), (80, 80), and (80, 120). We simulate 5000 samples from the non-diseased population and the diseased population. For each sample, *B* = 500 bootstrap samples are generated to calculate the bootstrap mean and variance in the BAC method. The proposed NP and NPAC intervals do not require bootstrap sampling, therefore, they are computationally easy as compared to the BAC interval.

**Fig 1:**
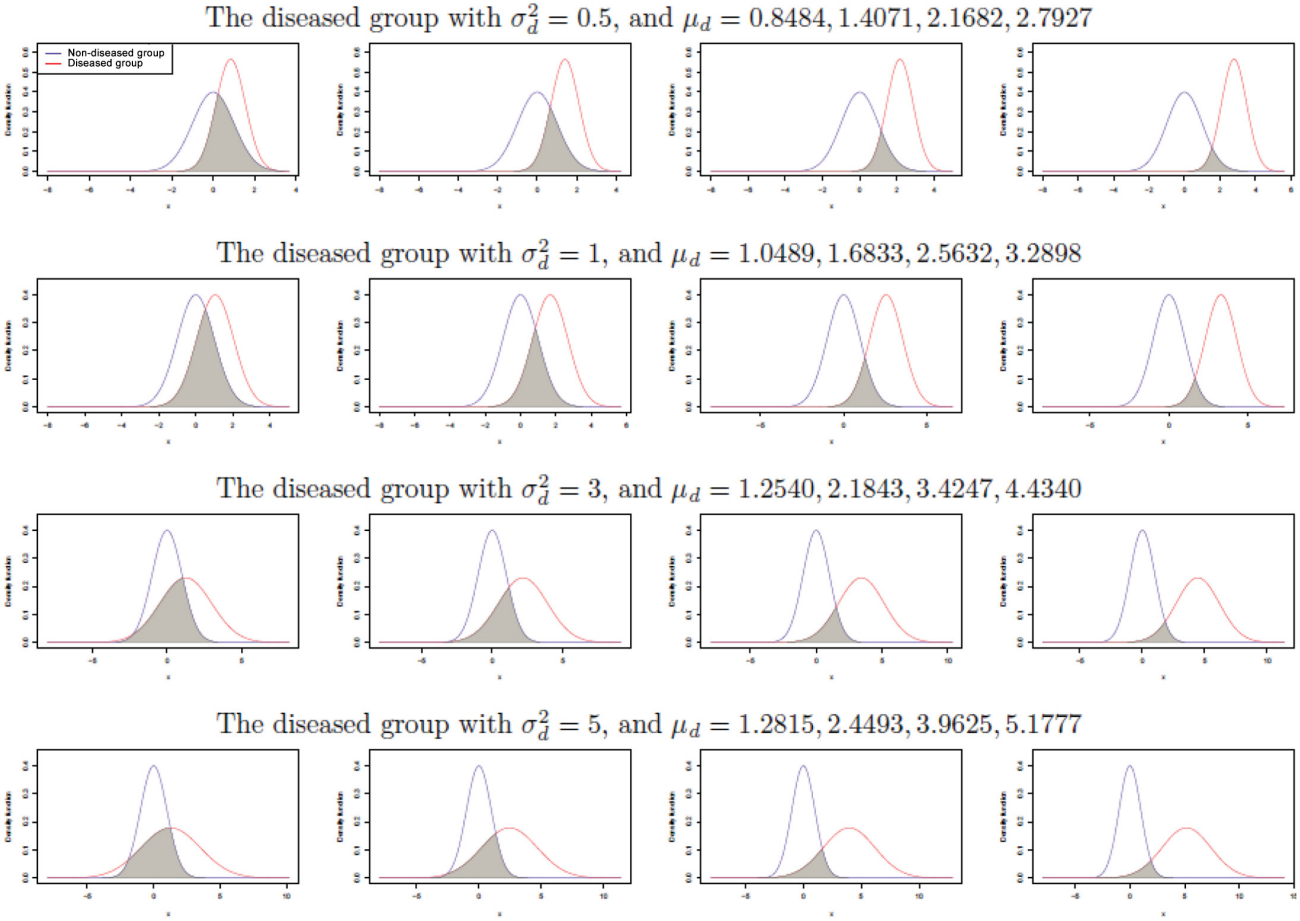
Density functions for a non-diseased group (a standard normal distribution) and a diseased group when the data is generated from two normal distributions as in Table 1.

We first compare the three methods with the same type of underlying distributions for the non-diseased group and the diseased group. The normal distribution is the most commonly used distribution in data analysis. The non-diseased group is assumed to follow a standard normal distribution, and the diseased group follows a normal distribution with parameters $N(\mu_{d},\sigma_{d}^{2})$, where $\sigma_{d}^{2}=0.5,1,3,$ and 5. For each given variance $\sigma_{d}^{2}$ in the diseased group, the associated *μ*_*d*_ values are computed in order to attain the pre-defined Youden Index values *J* = 0.4, 0.6, 0.8, and 0.9. There are a total of 16 combinations for the parameter settings considered in this comparison, and the detailed values of each parameter setting can be found in the first column of Table 1. Plots for density functions of the non-diseased group and the diseased group under these 16 parameter combinations, are presented in Fig 1. It can be seen that, for a given standard deviation, the overlapping area between the two distributions decreases as the difference in location between these two groups increases.

**Table 1:** Normal distributions: coverage probabilities and average widths when *m* = 20 in the non-diseased group at the 95% nominal level.

Parameter setting $\left(\mu_{d},\sigma_{d}^{2},J\right)$	n	BAC Coverage (width)	NP Coverage (width)	NPAC Coverage (width)
(0.8484, 0.5, 0.4)	20	0.898 (0.370)	0.933 (0.481)	0.994 (0.512)
	40	0.875 (0.348)	0.938 (0.432)	0.988 (0.451)
	60	0.863 (0.339)	0.927 (0.414)	0.985 (0.430)
(1.4071, 0.5, 0.6)	20	0.929 (0.318)	0.971 (0.432)	0.984 (0.478)
	40	0.918 (0.303)	0.960 (0.387)	0.990 (0.418)
	60	0.904 (0.293)	0.948 (0.369)	0.990 (0.397)
(2.1682, 0.5, 0.8)	20	0.877 (0.212)	0.997 (0.348)	0.852 (0.424)
	40	0.916 (0.206)	0.982 (0.306)	0.935 (0.362)
	60	0.921 (0.203)	0.977 (0.292)	0.944 (0.343)
(2.7927, 0.5, 0.9)	20	0.000 (0.118)	0.996 (0.289)	0.650 (0.390)
	40	0.238 (0.121)	0.992 (0.247)	0.750 (0.326)
	60	0.631 (0.120)	0.994 (0.234)	0.794 (0.306)
(1.0489, 1, 0.4)	20	0.877 (0.369)	0.915 (0.483)	0.993 (0.513)
	40	0.870 (0.338)	0.942 (0.428)	0.989 (0.450)
	60	0.854 (0.323)	0.931 (0.405)	0.985 (0.425)
(1.6833, 1, 0.6)	20	0.929 (0.316)	0.971 (0.432)	0.989 (0.478)
	40	0.918 (0.291)	0.960 (0.381)	0.991 (0.415)
	60	0.902 (0.280)	0.947 (0.360)	0.988 (0.392)
(2.5632, 1, 0.8)	20	0.893 (0.211)	0.997 (0.347)	0.867 (0.424)
	40	0.927 (0.200)	0.985 (0.302)	0.935 (0.360)
	60	0.926 (0.191)	0.982 (0.284)	0.932 (0.338)
(3.2898, 1, 0.9)	20	0.000 (0.116)	0.996 (0.288)	0.659 (0.390)
	40	0.175 (0.117)	0.995 (0.245)	0.723 (0.324)
	60	0.590 (0.114)	0.994 (0.230)	0.782 (0.304)
(1.254, 3, 0.4)	20	0.912 (0.370)	0.942 (0.481)	0.994 (0.511)
	40	0.906 (0.322)	0.951 (0.412)	0.988 (0.439)
	60	0.906 (0.297)	0.955 (0.380)	0.988 (0.408)
(2.1843, 3, 0.6)	20	0.936 (0.321)	0.975 (0.434)	0.982 (0.480)
	40	0.932 (0.281)	0.968 (0.372)	0.978 (0.409)
	60	0.932 (0.259)	0.965 (0.343)	0.981 (0.380)
(3.4247, 3, 0.8)	20	0.880 (0.219)	0.996 (0.351)	0.849 (0.426)
	40	0.915 (0.194)	0.988 (0.297)	0.908 (0.356)
	60	0.932 (0.180)	0.989 (0.277)	0.912 (0.332)
(4.434, 3, 0.9)	20	0.000 (0.124)	0.995 (0.291)	0.633 (0.392)
	40	0.076 (0.117)	0.994 (0.243)	0.643 (0.323)
	60	0.432 (0.112)	0.994 (0.229)	0.697 (0.302)
(1.2815, 5, 0.4)	20	0.930 (0.369)	0.957 (0.478)	0.996 (0.509)
	40	0.934 (0.313)	0.963 (0.402)	0.988 (0.431)
	60	0.929 (0.283)	0.964 (0.366)	0.986 (0.398)
(2.4493, 5, 0.6)	20	0.939 (0.325)	0.977 (0.436)	0.976 (0.480)
	40	0.940 (0.277)	0.972 (0.367)	0.978 (0.405)
	60	0.943 (0.251)	0.975 (0.337)	0.971 (0.376)
(3.9625, 5, 0.8)	20	0.863 (0.222)	0.995 (0.352)	0.842 (0.426)
	40	0.910 (0.195)	0.991 (0.297)	0.902 (0.356)
	60	0.915 (0.177)	0.991 (0.275)	0.888 (0.331)
(5.1777, 5, 0.9)	20	0.000 (0.127)	0.994 (0.292)	0.627 (0.392)
	40	0.048 (0.123)	0.994 (0.246)	0.573 (0.324)
	60	0.363 (0.112)	0.994 (0.228)	0.665 (0.301)

Tables 1–4 present the average coverage probabilities and average widths for the three methods when *m* = 20, 40, 60, and 80, respectively. The coverage probability is defined as the proportion of time that the computed confidence interval contains the pre-defined Youden Index value. Given the parameter setting and sample size in the non-diseased group, the average width decreases as the sample size increases in the diseased group for each method. The coverage probability for the BAC interval is often less than the nominal level. The coverage probability for the BAC interval performs very poorly when the sample sizes are small and the pre-defined *J* is large. The two newly proposed methods, the NP method and the NPAC method, generally have better coverage than the BAC method. The NPAC method does not perform as good as the NP method when the pre-defined *J* is large, such as 0.9. In such cases, the coverage probability based on the NPAC method could be as low as 57.3%. The NPAC method could have higher coverage probablity than the NP method when *J* is small, but the width of the interval would be much longer for the NPAC method. The NP method has satisfactory performance as compared to the other two methods.

**Table 2:** Normal distributions: coverage probabilities and average widths when *m* = 40 in the non-diseased group at the 95% nominal level.

Parameter setting $\left(\mu_{d},\sigma_{d}^{2},J\right)$	n	BAC Coverage (width)	NP Coverage (width)	NPAC Coverage (width)
(0.8484, 0.5, 0.4)	20	0.890 (0.328)	0.944 (0.419)	0.991 (0.444)
	40	0.873 (0.301)	0.932 (0.357)	0.983 (0.369)
	80	0.865 (0.279)	0.928 (0.322)	0.973 (0.328)
(1.4071, 0.5, 0.6)	20	0.926 (0.283)	0.963 (0.374)	0.982 (0.410)
	40	0.915 (0.264)	0.947 (0.318)	0.980 (0.337)
	80	0.914 (0.246)	0.949 (0.287)	0.986 (0.299)
(2.1682, 0.5, 0.8)	20	0.924 (0.196)	0.987 (0.299)	0.918 (0.358)
	40	0.942 (0.188)	0.960 (0.247)	0.952 (0.282)
	80	0.943 (0.175)	0.972 (0.219)	0.961 (0.244)
(2.7927, 0.5, 0.9)	20	0.110 (0.117)	0.995 (0.244)	0.661 (0.324)
	40	0.840 (0.120)	0.994 (0.193)	0.850 (0.244)
	80	0.919 (0.115)	0.977 (0.168)	0.871 (0.206)
(1.0489, 1, 0.4)	20	0.870 (0.339)	0.942 (0.429)	0.989 (0.450)
	40	0.861 (0.303)	0.929 (0.361)	0.980 (0.373)
	80	0.864 (0.272)	0.936 (0.318)	0.976 (0.326)
(1.6833, 1, 0.6)	20	0.912 (0.292)	0.960 (0.381)	0.987 (0.415)
	40	0.912 (0.264)	0.950 (0.319)	0.984 (0.338)
	80	0.909 (0.237)	0.944 (0.280)	0.984 (0.294)
(2.5632, 1, 0.8)	20	0.923 (0.199)	0.984 (0.302)	0.932 (0.360)
	40	0.943 (0.187)	0.961 (0.246)	0.958 (0.282)
	80	0.937 (0.169)	0.970 (0.215)	0.954 (0.241)
(3.2898, 1, 0.9)	20	0.158 (0.118)	0.993 (0.245)	0.711 (0.325)
	40	0.840 (0.118)	0.996 (0.192)	0.855 (0.243)
	80	0.921 (0.110)	0.985 (0.165)	0.864 (0.204)
(1.254, 3, 0.4)	20	0.879 (0.352)	0.937 (0.431)	0.988 (0.450)
	40	0.900 (0.299)	0.943 (0.355)	0.984 (0.367)
	80	0.900 (0.252)	0.945 (0.297)	0.977 (0.309)
(2.1843, 3, 0.6)	20	0.917 (0.308)	0.958 (0.390)	0.986 (0.420)
	40	0.921 (0.264)	0.954 (0.318)	0.979 (0.337)
	80	0.924 (0.224)	0.951 (0.268)	0.979 (0.284)
(3.4247, 3, 0.8)	20	0.916 (0.212)	0.976 (0.308)	0.939 (0.364)
	40	0.948 (0.189)	0.963 (0.248)	0.956 (0.283)
	80	0.945 (0.161)	0.976 (0.208)	0.945 (0.236)
(4.434, 3, 0.9)	20	0.285 (0.125)	0.994 (0.248)	0.764 (0.327)
	40	0.847 (0.122)	0.995 (0.193)	0.845 (0.244)
	80	0.903 (0.107)	0.986 (0.162)	0.842 (0.202)
(1.2815, 5, 0.4)	20	0.899 (0.356)	0.941 (0.429)	0.992 (0.447)
	40	0.910 (0.296)	0.945 (0.349)	0.986 (0.362)
	80	0.920 (0.242)	0.954 (0.285)	0.982 (0.299)
(2.4493, 5, 0.6)	20	0.922 (0.317)	0.958 (0.393)	0.984 (0.422)
	40	0.938 (0.265)	0.960 (0.317)	0.976 (0.336)
	80	0.931 (0.218)	0.957 (0.261)	0.974 (0.279)
(3.9625, 5, 0.8)	20	0.919 (0.221)	0.980 (0.313)	0.935 (0.367)
	40	0.939 (0.192)	0.964 (0.249)	0.945 (0.284)
	80	0.947 (0.159)	0.979 (0.207)	0.936 (0.235)
(5.1777, 5, 0.9)	20	0.347 (0.131)	0.990 (0.251)	0.777 (0.329)
	40	0.826 (0.125)	0.992 (0.195)	0.832 (0.245)
	80	0.896 (0.107)	0.990 (0.162)	0.828 (0.201)

**Table 3:** Normal distributions: coverage probabilities and average widths when *m* = 60 in the non-diseased group at the 95% nominal level.

Parameter setting $\left(\mu_{d},\sigma_{d}^{2},J\right)$	n	BAC Coverage (width)	NP Coverage (width)	NPAC Coverage (width)
(0.8484, 0.5, 0.4)	30	0.869 (0.288)	0.932 (0.349)	0.979 (0.364)
	60	0.873 (0.260)	0.932 (0.298)	0.977 (0.305)
	90	0.876 (0.247)	0.929 (0.279)	0.969 (0.283)
(1.4071, 0.5, 0.6)	30	0.914 (0.252)	0.952 (0.311)	0.982 (0.333)
	60	0.911 (0.229)	0.941 (0.265)	0.980 (0.276)
	90	0.912 (0.218)	0.940 (0.247)	0.977 (0.255)
(2.1682, 0.5, 0.8)	30	0.939 (0.178)	0.980 (0.244)	0.940 (0.282)
	60	0.937 (0.165)	0.965 (0.202)	0.966 (0.224)
	90	0.936 (0.158)	0.957 (0.188)	0.960 (0.205)
(2.7927, 0.5, 0.9)	30	0.835 (0.114)	0.993 (0.192)	0.829 (0.246)
	60	0.939 (0.111)	0.991 (0.154)	0.843 (0.186)
	90	0.936 (0.106)	0.972 (0.141)	0.905 (0.167)
(1.0489, 1, 0.4)	30	0.856 (0.300)	0.933 (0.361)	0.982 (0.372)
	60	0.855 (0.262)	0.928 (0.302)	0.974 (0.308)
	90	0.864 (0.244)	0.928 (0.279)	0.962 (0.283)
(1.6833, 1, 0.6)	30	0.906 (0.261)	0.954 (0.319)	0.984 (0.339)
	60	0.910 (0.229)	0.940 (0.266)	0.976 (0.277)
	90	0.899 (0.214)	0.939 (0.244)	0.982 (0.253)
(2.5632, 1, 0.8)	30	0.935 (0.183)	0.971 (0.247)	0.949 (0.284)
	60	0.941 (0.164)	0.967 (0.202)	0.964 (0.224)
	90	0.941 (0.154)	0.960 (0.185)	0.966 (0.203)
(3.2898, 1, 0.9)	30	0.845 (0.116)	0.993 (0.194)	0.847 (0.247)
	60	0.937 (0.110)	0.985 (0.153)	0.837 (0.186)
	90	0.942 (0.102)	0.976 (0.138)	0.914 (0.165)
(1.254, 3, 0.4)	30	0.879 (0.311)	0.930 (0.364)	0.978 (0.373)
	60	0.898 (0.257)	0.940 (0.295)	0.982 (0.302)
	90	0.897 (0.231)	0.939 (0.262)	0.972 (0.269)
(2.1843, 3, 0.6)	30	0.911 (0.275)	0.954 (0.327)	0.984 (0.344)
	60	0.923 (0.229)	0.947 (0.264)	0.980 (0.275)
	90	0.917 (0.205)	0.947 (0.235)	0.976 (0.246)
(3.4247, 3, 0.8)	30	0.933 (0.196)	0.972 (0.255)	0.946 (0.290)
	60	0.943 (0.166)	0.972 (0.203)	0.965 (0.224)
	90	0.945 (0.150)	0.961 (0.181)	0.963 (0.199)
(4.434, 3, 0.9)	30	0.847 (0.123)	0.992 (0.198)	0.851 (0.250)
	60	0.929 (0.112)	0.985 (0.154)	0.826 (0.187)
	90	0.940 (0.102)	0.984 (0.137)	0.898 (0.164)
(1.2815, 5, 0.4)	30	0.906 (0.313)	0.940 (0.361)	0.981 (0.370)
	60	0.919 (0.253)	0.946 (0.288)	0.979 (0.296)
	90	0.912 (0.223)	0.942 (0.252)	0.979 (0.261)
(2.4493, 5, 0.6)	30	0.930 (0.282)	0.961 (0.330)	0.984 (0.346)
	60	0.929 (0.229)	0.948 (0.263)	0.976 (0.274)
	90	0.928 (0.202)	0.951 (0.231)	0.975 (0.242)
(3.9625, 5, 0.8)	30	0.937 (0.202)	0.975 (0.258)	0.948 (0.292)
	60	0.945 (0.168)	0.968 (0.203)	0.959 (0.224)
	90	0.951 (0.149)	0.964 (0.180)	0.957 (0.198)
(5.1777, 5, 0.9)	30	0.842 (0.130)	0.991 (0.201)	0.841 (0.252)
	60	0.931 (0.114)	0.990 (0.155)	0.822 (0.187)
	90	0.933 (0.102)	0.983 (0.138)	0.886 (0.164)

**Table 4:** Normal distributions: coverage probabilities and average widths when *m* = 80 in the non-diseased group at the 95% nominal level.

Parameter setting $\left(\mu_{d},\sigma_{d}^{2},J\right)$	n	BAC Coverage (width)	NP Coverage (width)	NPAC Coverage (width)
(0.8484, 0.5, 0.4)	60	0.880 (0.243)	0.929 (0.277)	0.968 (0.283)
	80	0.880 (0.233)	0.931 (0.261)	0.969 (0.265)
	120	0.880 (0.220)	0.933 (0.245)	0.964 (0.247)
(1.4071, 0.5, 0.6)	60	0.920 (0.214)	0.949 (0.246)	0.978 (0.256)
	80	0.919 (0.206)	0.954 (0.232)	0.981 (0.239)
	120	0.899 (0.195)	0.932 (0.217)	0.974 (0.222)
(2.1682, 0.5, 0.8)	60	0.947 (0.155)	0.964 (0.187)	0.964 (0.206)
	80	0.937 (0.150)	0.959 (0.176)	0.972 (0.191)
	120	0.940 (0.142)	0.956 (0.164)	0.966 (0.175)
(2.7927, 0.5, 0.9)	60	0.941 (0.104)	0.978 (0.142)	0.893 (0.170)
	80	0.951 (0.102)	0.985 (0.132)	0.900 (0.155)
	120	0.939 (0.097)	0.968 (0.122)	0.926 (0.140)
(1.0489, 1, 0.4)	60	0.861 (0.249)	0.920 (0.285)	0.966 (0.289)
	80	0.868 (0.235)	0.928 (0.266)	0.966 (0.269)
	120	0.865 (0.218)	0.923 (0.244)	0.960 (0.247)
(1.6833, 1, 0.6)	60	0.898 (0.218)	0.936 (0.250)	0.981 (0.259)
	80	0.903 (0.206)	0.943 (0.233)	0.976 (0.240)
	120	0.904 (0.191)	0.940 (0.214)	0.974 (0.220)
(2.5632, 1, 0.8)	60	0.941 (0.157)	0.959 (0.190)	0.965 (0.208)
	80	0.933 (0.149)	0.960 (0.176)	0.972 (0.191)
	120	0.937 (0.139)	0.955 (0.162)	0.969 (0.173)
(3.2898, 1, 0.9)	60	0.942 (0.105)	0.982 (0.143)	0.889 (0.171)
	80	0.944 (0.101)	0.981 (0.131)	0.904 (0.154)
	120	0.944 (0.095)	0.975 (0.120)	0.924 (0.139)
(1.254, 3, 0.4)	60	0.895 (0.250)	0.934 (0.281)	0.973 (0.285)
	80	0.886 (0.229)	0.930 (0.258)	0.968 (0.262)
	120	0.889 (0.205)	0.933 (0.228)	0.967 (0.233)
(2.1843, 3, 0.6)	60	0.916 (0.222)	0.943 (0.252)	0.978 (0.260)
	80	0.912 (0.205)	0.944 (0.231)	0.976 (0.238)
	120	0.921 (0.183)	0.949 (0.206)	0.979 (0.212)
(3.4247, 3, 0.8)	60	0.944 (0.162)	0.966 (0.193)	0.960 (0.211)
	80	0.938 (0.150)	0.961 (0.177)	0.964 (0.191)
	120	0.934 (0.135)	0.953 (0.157)	0.964 (0.170)
(4.434, 3, 0.9)	60	0.936 (0.110)	0.975 (0.145)	0.880 (0.172)
	80	0.946 (0.103)	0.981 (0.133)	0.892 (0.155)
	120	0.947 (0.092)	0.975 (0.118)	0.919 (0.137)
(1.2815, 5, 0.4)	60	0.909 (0.247)	0.935 (0.275)	0.973 (0.280)
	80	0.913 (0.225)	0.946 (0.250)	0.975 (0.255)
	120	0.909 (0.197)	0.942 (0.219)	0.974 (0.225)
(2.4493, 5, 0.6)	60	0.925 (0.224)	0.945 (0.251)	0.977 (0.260)
	80	0.927 (0.204)	0.948 (0.229)	0.979 (0.237)
	120	0.930 (0.180)	0.950 (0.201)	0.976 (0.209)
(3.9625, 5, 0.8)	60	0.944 (0.164)	0.964 (0.194)	0.958 (0.211)
	80	0.941 (0.151)	0.960 (0.177)	0.964 (0.191)
	120	0.948 (0.134)	0.961 (0.156)	0.959 (0.169)
(5.1777, 5, 0.9)	60	0.920 (0.112)	0.975 (0.146)	0.878 (0.173)
	80	0.948 (0.104)	0.983 (0.133)	0.886 (0.155)
	120	0.947 (0.093)	0.981 (0.118)	0.912 (0.136)

The three methods are also compared under gamma distributions, Γ(*κ*, *θ*), with the probability density function

$$\begin{array}{c} \Gamma \left(x;\kappa ,\theta \right)=\frac{\theta^{\kappa }}{\Gamma (\kappa )}x^{\kappa -1}e^{-\theta x}. \end{array}$$

The expectation of the gamma distribution is *κ*/*θ*. The non-diseased group follows a gamma distribution with parameters *κ*_*X*_ = 1.5 and *θ*_*X*_ = 1, and the diseased group with *κ*_*Y*_ = (1.5, 2, 2.5, 3) and *θ*_*Y*_, where *θ*_*Y*_ is calculated in order to achieve the Youdex Index *J* = 0.4, 0.6, 0.8, and 0.9, for each *κ*_*Y*_. 16 total parameter settings are given in the first column of Table 5. Tables 5–8 show the coverage probabilities and average widths under the gamma distributions for *m* = 20, 40, 60, and 80, respectively. We observe similar results in normal distributions.

**Table 5:** Gamma distributions: coverage probabilities and average widths when *m* = 20 in the non-diseased group at the 95% nominal level.

Parameter setting (*κ*_*Y*_, *θ*_*Y*_, *J*)	n	BAC Coverage (width)	NP Coverage (width)	NPAC Coverage (width)
(1.5, 0.4028, 0.4)	20	0.879 (0.369)	0.924 (0.483)	0.992 (0.513)
	40	0.877 (0.329)	0.939 (0.421)	0.992 (0.445)
	60	0.880 (0.310)	0.946 (0.394)	0.992 (0.418)
(1.5, 0.2295, 0.6)	20	0.935 (0.319)	0.976 (0.434)	0.985 (0.479)
	40	0.920 (0.280)	0.964 (0.372)	0.981 (0.409)
	60	0.926 (0.261)	0.959 (0.345)	0.982 (0.381)
(1.5, 0.1022, 0.8)	20	0.869 (0.219)	0.995 (0.351)	0.849 (0.426)
	40	0.911 (0.194)	0.991 (0.298)	0.900 (0.357)
	60	0.919 (0.178)	0.992 (0.276)	0.901 (0.332)
(1.5, 0.0505, 0.9)	20	0.000 (0.129)	0.995 (0.292)	0.630 (0.392)
	40	0.041 (0.123)	0.994 (0.245)	0.567 (0.323)
	60	0.328 (0.113)	0.995 (0.228)	0.656 (0.301)
(2, 0.6016, 0.4)	20	0.882 (0.368)	0.922 (0.483)	0.996 (0.513)
	40	0.870 (0.334)	0.939 (0.426)	0.991 (0.448)
	60	0.858 (0.318)	0.937 (0.402)	0.989 (0.423)
(2, 0.3616, 0.6)	20	0.928 (0.316)	0.966 (0.432)	0.988 (0.479)
	40	0.924 (0.285)	0.964 (0.376)	0.986 (0.412)
	60	0.916 (0.267)	0.952 (0.351)	0.986 (0.385)
(2, 0.1769, 0.8)	20	0.868 (0.214)	0.996 (0.349)	0.853 (0.425)
	40	0.924 (0.194)	0.990 (0.298)	0.915 (0.357)
	60	0.928 (0.179)	0.990 (0.276)	0.919 (0.332)
(2, 0.0963, 0.9)	20	0.000 (0.125)	0.994 (0.291)	0.638 (0.392)
	40	0.063 (0.120)	0.994 (0.245)	0.610 (0.324)
	60	0.396 (0.112)	0.995 (0.228)	0.685 (0.301)
(2.5, 0.8189, 0.4)	20	0.878 (0.368)	0.917 (0.482)	0.993 (0.513)
	40	0.866 (0.339)	0.932 (0.428)	0.989 (0.449)
	60	0.860 (0.325)	0.938 (0.407)	0.989 (0.426)
(2.5, 0.5064, 0.6)	20	0.930 (0.315)	0.969 (0.432)	0.989 (0.478)
	40	0.919 (0.288)	0.962 (0.379)	0.984 (0.413)
	60	0.913 (0.274)	0.951 (0.357)	0.989 (0.389)
(2.5, 0.2619, 0.8)	20	0.876 (0.213)	0.996 (0.349)	0.851 (0.425)
	40	0.922 (0.196)	0.986 (0.300)	0.912 (0.358)
	60	0.935 (0.184)	0.987 (0.280)	0.925 (0.335)
(2.5, 0.151, 0.9)	20	0.000 (0.118)	0.997 (0.288)	0.646 (0.390)
	40	0.086 (0.119)	0.995 (0.245)	0.629 (0.324)
	60	0.454 (0.111)	0.993 (0.228)	0.732 (0.301)
(3, 1.0522, 0.4)	20	0.886 (0.369)	0.922 (0.482)	0.995 (0.513)
	40	0.870 (0.342)	0.935 (0.431)	0.987 (0.451)
	60	0.853 (0.330)	0.928 (0.410)	0.985 (0.428)
(3, 0.6614, 0.6)	20	0.931 (0.317)	0.973 (0.432)	0.985 (0.479)
	40	0.911 (0.291)	0.954 (0.380)	0.987 (0.414)
	60	0.907 (0.278)	0.950 (0.360)	0.987 (0.391)
(3, 0.3547, 0.8)	20	0.884 (0.212)	0.997 (0.348)	0.858 (0.424)
	40	0.925 (0.196)	0.987 (0.300)	0.929 (0.359)
	60	0.932 (0.186)	0.984 (0.282)	0.924 (0.336)
(3, 0.2124, 0.9)	20	0.000 (0.118)	0.994 (0.288)	0.669 (0.390)
	40	0.108 (0.118)	0.996 (0.245)	0.660 (0.324)
	60	0.490 (0.113)	0.994 (0.230)	0.724 (0.303)

**Table 6:** Gamma distributions: coverage probabilities and average widths when *m* = 40 in the non-diseased group at the 95% nominal level.

Parameter setting (*κ*_*Y*_, *θ*_*Y*_, *J*)	n	BAC Coverage (width)	NP Coverage (width)	NPAC Coverage (width)
(1.5, 0.4028, 0.4)	20	0.865 (0.346)	0.935 (0.432)	0.987 (0.451)
	40	0.884 (0.301)	0.942 (0.359)	0.983 (0.371)
	80	0.870 (0.262)	0.931 (0.309)	0.977 (0.318)
(1.5, 0.2295, 0.6)	20	0.917 (0.306)	0.958 (0.389)	0.984 (0.419)
	40	0.925 (0.264)	0.954 (0.319)	0.980 (0.338)
	80	0.919 (0.224)	0.949 (0.269)	0.983 (0.285)
(1.5, 0.1022, 0.8)	20	0.911 (0.215)	0.974 (0.310)	0.940 (0.365)
	40	0.937 (0.189)	0.961 (0.247)	0.953 (0.282)
	80	0.938 (0.160)	0.974 (0.207)	0.937 (0.235)
(1.5, 0.0505, 0.9)	20	0.351 (0.133)	0.990 (0.253)	0.780 (0.330)
	40	0.825 (0.127)	0.992 (0.196)	0.826 (0.245)
	80	0.882 (0.106)	0.989 (0.161)	0.822 (0.200)
(2, 0.6016, 0.4)	20	0.859 (0.341)	0.930 (0.429)	0.989 (0.450)
	40	0.863 (0.302)	0.930 (0.362)	0.979 (0.373)
	80	0.860 (0.268)	0.935 (0.315)	0.978 (0.323)
(2, 0.3616, 0.6)	20	0.912 (0.299)	0.957 (0.385)	0.988 (0.417)
	40	0.919 (0.263)	0.956 (0.319)	0.981 (0.338)
	80	0.910 (0.230)	0.947 (0.274)	0.984 (0.289)
(2, 0.1769, 0.8)	20	0.916 (0.211)	0.979 (0.309)	0.930 (0.364)
	40	0.941 (0.189)	0.965 (0.248)	0.954 (0.283)
	80	0.948 (0.162)	0.978 (0.209)	0.946 (0.237)
(2, 0.0963, 0.9)	20	0.310 (0.128)	0.990 (0.251)	0.758 (0.328)
	40	0.819 (0.122)	0.993 (0.193)	0.835 (0.244)
	80	0.902 (0.106)	0.990 (0.162)	0.835 (0.201)
(2.5, 0.8189, 0.4)	20	0.857 (0.336)	0.933 (0.427)	0.991 (0.449)
	40	0.871 (0.302)	0.942 (0.362)	0.985 (0.373)
	80	0.866 (0.273)	0.935 (0.319)	0.978 (0.326)
(2.5, 0.5064, 0.6)	20	0.909 (0.295)	0.955 (0.383)	0.982 (0.416)
	40	0.907 (0.263)	0.947 (0.319)	0.978 (0.338)
	80	0.903 (0.233)	0.947 (0.277)	0.984 (0.292)
(2.5, 0.2619, 0.8)	20	0.914 (0.204)	0.975 (0.305)	0.935 (0.362)
	40	0.937 (0.187)	0.956 (0.247)	0.952 (0.282)
	80	0.937 (0.163)	0.969 (0.211)	0.956 (0.238)
(2.5, 0.151, 0.9)	20	0.274 (0.122)	0.991 (0.248)	0.768 (0.327)
	40	0.837 (0.121)	0.995 (0.193)	0.845 (0.244)
	80	0.910 (0.107)	0.989 (0.163)	0.845 (0.202)
(3, 1.0522, 0.4)	20	0.872 (0.334)	0.941 (0.426)	0.990 (0.448)
	40	0.873 (0.302)	0.939 (0.360)	0.982 (0.372)
	80	0.865 (0.275)	0.929 (0.321)	0.972 (0.328)
(3, 0.6614, 0.6)	20	0.911 (0.290)	0.959 (0.380)	0.988 (0.414)
	40	0.918 (0.263)	0.953 (0.319)	0.985 (0.338)
	80	0.897 (0.237)	0.939 (0.280)	0.982 (0.294)
(3, 0.3547, 0.8)	20	0.918 (0.204)	0.983 (0.304)	0.928 (0.362)
	40	0.942 (0.186)	0.959 (0.246)	0.964 (0.282)
	80	0.940 (0.165)	0.974 (0.212)	0.951 (0.239)
(3, 0.2124, 0.9)	20	0.241 (0.120)	0.992 (0.247)	0.754 (0.326)
	40	0.841 (0.119)	0.995 (0.192)	0.854 (0.243)
	80	0.913 (0.107)	0.984 (0.163)	0.855 (0.202)

**Table 7:** Gamma distributions: coverage probabilities and average widths when *m* = 60 in the non-diseased group at the 95% nominal level.

Parameter setting (*κ*_*Y*_, *θ*_*Y*_, *J*)	n	BAC Coverage (width)	NP Coverage (width)	NPAC Coverage (width)
(1.5, 0.4028, 0.4)	30	0.869 (0.307)	0.936 (0.364)	0.979 (0.374)
	60	0.870 (0.260)	0.928 (0.300)	0.973 (0.306)
	90	0.871 (0.237)	0.930 (0.271)	0.972 (0.277)
(1.5, 0.2295, 0.6)	30	0.910 (0.272)	0.955 (0.326)	0.984 (0.344)
	60	0.915 (0.229)	0.947 (0.265)	0.981 (0.276)
	90	0.905 (0.206)	0.933 (0.237)	0.976 (0.247)
(1.5, 0.1022, 0.8)	30	0.934 (0.199)	0.970 (0.257)	0.950 (0.291)
	60	0.947 (0.167)	0.970 (0.203)	0.962 (0.224)
	90	0.945 (0.149)	0.960 (0.180)	0.957 (0.198)
(1.5, 0.0505, 0.9)	30	0.846 (0.132)	0.990 (0.203)	0.845 (0.253)
	60	0.929 (0.114)	0.992 (0.155)	0.833 (0.187)
	90	0.928 (0.102)	0.984 (0.138)	0.877 (0.164)
(2, 0.6016, 0.4)	30	0.858 (0.303)	0.932 (0.362)	0.977 (0.374)
	60	0.866 (0.261)	0.936 (0.303)	0.978 (0.309)
	90	0.861 (0.241)	0.927 (0.277)	0.967 (0.282)
(2, 0.3616, 0.6)	30	0.916 (0.267)	0.953 (0.324)	0.985 (0.342)
	60	0.906 (0.228)	0.936 (0.265)	0.972 (0.276)
	90	0.918 (0.209)	0.947 (0.240)	0.979 (0.250)
(2, 0.1769, 0.8)	30	0.930 (0.194)	0.972 (0.254)	0.944 (0.289)
	60	0.943 (0.166)	0.969 (0.203)	0.963 (0.224)
	90	0.942 (0.150)	0.958 (0.182)	0.958 (0.200)
(2, 0.0963, 0.9)	30	0.838 (0.126)	0.992 (0.200)	0.841 (0.251)
	60	0.929 (0.112)	0.987 (0.154)	0.832 (0.186)
	90	0.937 (0.102)	0.986 (0.137)	0.896 (0.164)
(2.5, 0.8189, 0.4)	30	0.857 (0.298)	0.932 (0.360)	0.981 (0.372)
	60	0.873 (0.262)	0.934 (0.303)	0.973 (0.309)
	90	0.860 (0.244)	0.929 (0.279)	0.969 (0.283)
(2.5, 0.5064, 0.6)	30	0.910 (0.264)	0.952 (0.321)	0.983 (0.341)
	60	0.906 (0.229)	0.944 (0.266)	0.981 (0.277)
	90	0.900 (0.211)	0.941 (0.242)	0.980 (0.251)
(2.5, 0.2619, 0.8)	30	0.937 (0.189)	0.972 (0.251)	0.952 (0.287)
	60	0.942 (0.165)	0.969 (0.203)	0.964 (0.224)
	90	0.937 (0.151)	0.956 (0.182)	0.962 (0.200)
(2.5, 0.151, 0.9)	30	0.851 (0.122)	0.992 (0.198)	0.858 (0.250)
	60	0.938 (0.112)	0.991 (0.154)	0.829 (0.187)
	90	0.940 (0.101)	0.985 (0.137)	0.902 (0.164)
(3, 1.0522, 0.4)	30	0.855 (0.294)	0.930 (0.356)	0.977 (0.369)
	60	0.863 (0.261)	0.930 (0.302)	0.975 (0.308)
	90	0.863 (0.245)	0.927 (0.279)	0.969 (0.284)
(3, 0.6614, 0.6)	30	0.916 (0.261)	0.960 (0.319)	0.986 (0.339)
	60	0.904 (0.229)	0.946 (0.266)	0.979 (0.277)
	90	0.905 (0.213)	0.942 (0.244)	0.980 (0.253)
(3, 0.3547, 0.8)	30	0.940 (0.187)	0.975 (0.250)	0.955 (0.286)
	60	0.943 (0.165)	0.974 (0.203)	0.966 (0.224)
	90	0.939 (0.152)	0.961 (0.184)	0.963 (0.201)
(3, 0.2124, 0.9)	30	0.854 (0.120)	0.992 (0.196)	0.860 (0.249)
	60	0.935 (0.110)	0.986 (0.154)	0.834 (0.186)
	90	0.935 (0.101)	0.980 (0.137)	0.903 (0.164)

**Table 8:** Gamma distributions: coverage probabilities and average widths when *m* = 80 in the non-diseased group at the 95% nominal level.

Parameter setting (*κ*_*Y*_, *θ*_*Y*_, *J*)	n	BAC Coverage (width)	NP Coverage (width)	NPAC Coverage (width)
(1.5, 0.4028, 0.4)	60	0.867 (0.250)	0.918 (0.284)	0.967 (0.289)
	80	0.870 (0.233)	0.927 (0.263)	0.965 (0.267)
	120	0.876 (0.211)	0.932 (0.237)	0.965 (0.241)
(1.5, 0.2295, 0.6)	60	0.910 (0.221)	0.942 (0.252)	0.977 (0.260)
	80	0.912 (0.205)	0.948 (0.232)	0.978 (0.239)
	120	0.911 (0.184)	0.943 (0.207)	0.974 (0.213)
(1.5, 0.1022, 0.8)	60	0.942 (0.164)	0.964 (0.194)	0.963 (0.211)
	80	0.947 (0.151)	0.968 (0.177)	0.968 (0.191)
	120	0.943 (0.134)	0.961 (0.156)	0.959 (0.169)
(1.5, 0.0505, 0.9)	60	0.925 (0.113)	0.979 (0.147)	0.879 (0.174)
	80	0.940 (0.105)	0.980 (0.133)	0.885 (0.156)
	120	0.950 (0.092)	0.984 (0.117)	0.916 (0.136)
(2, 0.6016, 0.4)	60	0.859 (0.249)	0.926 (0.285)	0.967 (0.290)
	80	0.867 (0.234)	0.929 (0.265)	0.970 (0.269)
	120	0.863 (0.215)	0.928 (0.242)	0.965 (0.245)
(2, 0.3616, 0.6)	60	0.910 (0.220)	0.945 (0.251)	0.979 (0.260)
	80	0.907 (0.205)	0.946 (0.233)	0.979 (0.240)
	120	0.909 (0.187)	0.944 (0.210)	0.977 (0.216)
(2, 0.1769, 0.8)	60	0.932 (0.161)	0.958 (0.192)	0.962 (0.210)
	80	0.940 (0.150)	0.966 (0.176)	0.969 (0.191)
	120	0.935 (0.134)	0.953 (0.157)	0.966 (0.170)
(2, 0.0963, 0.9)	60	0.937 (0.111)	0.980 (0.146)	0.874 (0.173)
	80	0.942 (0.103)	0.983 (0.132)	0.900 (0.155)
	120	0.945 (0.092)	0.978 (0.118)	0.913 (0.136)
(2.5, 0.8189, 0.4)	60	0.857 (0.248)	0.923 (0.284)	0.970 (0.289)
	80	0.857 (0.234)	0.925 (0.265)	0.966 (0.269)
	120	0.869 (0.218)	0.933 (0.245)	0.964 (0.248)
(2.5, 0.5064, 0.6)	60	0.906 (0.218)	0.942 (0.250)	0.981 (0.259)
	80	0.907 (0.205)	0.951 (0.233)	0.979 (0.240)
	120	0.893 (0.189)	0.935 (0.212)	0.971 (0.218)
(2.5, 0.2619, 0.8)	60	0.933 (0.159)	0.957 (0.191)	0.963 (0.209)
	80	0.945 (0.149)	0.968 (0.176)	0.972 (0.191)
	120	0.940 (0.136)	0.956 (0.159)	0.967 (0.171)
(2.5, 0.151, 0.9)	60	0.930 (0.109)	0.979 (0.145)	0.891 (0.172)
	80	0.940 (0.102)	0.981 (0.133)	0.892 (0.155)
	120	0.943 (0.092)	0.978 (0.118)	0.920 (0.136)
(3, 1.0522, 0.4)	60	0.863 (0.247)	0.925 (0.282)	0.973 (0.288)
	80	0.872 (0.234)	0.930 (0.264)	0.967 (0.268)
	120	0.867 (0.219)	0.927 (0.246)	0.961 (0.248)
(3, 0.6614, 0.6)	60	0.916 (0.217)	0.950 (0.250)	0.982 (0.259)
	80	0.907 (0.205)	0.953 (0.233)	0.980 (0.240)
	120	0.902 (0.190)	0.941 (0.214)	0.971 (0.220)
(3, 0.3547, 0.8)	60	0.935 (0.158)	0.959 (0.190)	0.965 (0.209)
	80	0.939 (0.149)	0.969 (0.176)	0.973 (0.191)
	120	0.941 (0.137)	0.956 (0.160)	0.963 (0.172)
(3, 0.2124, 0.9)	60	0.937 (0.107)	0.980 (0.144)	0.893 (0.172)
	80	0.944 (0.101)	0.982 (0.132)	0.899 (0.155)
	120	0.948 (0.093)	0.976 (0.119)	0.926 (0.137)

It is totally possible that the two groups do not follow the same type of distribution. For this reason, the three methods are compared with different distributions for the non-diseased group and the diseased group. The first case is that the non-diseased group follows a *t* distribution with *df* = 5, and the diseased group follows a normal distribution, $N(\mu_{Y},\sigma_{Y}^{2})$, with *σ*_*Y*_ = 1. The mean values *μ*_*Y*_ in the diseased group are calculated as 1.08, 1.75, 2.74, and 3.62 in order to attain *J* = 0.4, 0.6, 0.8, and 0.9, respectively. The coverage probabilities and average widths with various sample sizes are presented in Table 9. The coverage probabilities of the BAC method are much smaller than the nominal level for small *J*, such as 0.4, even with medium to large sample sizes. The NPAC method is very conservative for small *J*, and the average widths are longer than the other two methods in such cases. In addition, the NPAC generally has shorter coverage when *J* is large, e.g., 0.9. Overall, the NP method is robust to the *J* values and it has much better overall coverage property than the other two methods.

**Table 9:** Mixed distribution (t distribution and normal distribution): coverage probabilities and average widths at the 95% nominal level.

*μ*_*Y*_	*J*	m	n	BAC Coverage (width)	NP Coverage (width)	NPAC Coverage (width)
1.08	0.4	20	20	0.887 (0.372)	0.925 (0.483)	0.993 (0.514)
		20	40	0.873 (0.345)	0.941 (0.432)	0.990 (0.452)
		20	60	0.865 (0.331)	0.931 (0.409)	0.987 (0.428)
1.75	0.6	20	20	0.934 (0.321)	0.974 (0.434)	0.984 (0.480)
		20	40	0.922 (0.300)	0.961 (0.385)	0.986 (0.417)
		20	80	0.913 (0.290)	0.955 (0.366)	0.985 (0.396)
2.74	0.8	20	30	0.872 (0.217)	0.996 (0.349)	0.851 (0.425)
		20	60	0.918 (0.210)	0.983 (0.307)	0.926 (0.363)
		20	90	0.927 (0.203)	0.975 (0.291)	0.941 (0.342)
3.62	0.9	20	60	0.000 (0.127)	0.994 (0.292)	0.623 (0.392)
		20	80	0.257 (0.127)	0.989 (0.249)	0.737 (0.328)
		20	120	0.643 (0.127)	0.991 (0.237)	0.786 (0.309)
1.08	0.4	40	20	0.877 (0.339)	0.938 (0.428)	0.990 (0.450)
		40	40	0.878 (0.305)	0.940 (0.363)	0.980 (0.373)
		40	60	0.874 (0.277)	0.933 (0.321)	0.974 (0.328)
1.75	0.6	40	20	0.922 (0.292)	0.963 (0.380)	0.982 (0.414)
		40	40	0.923 (0.267)	0.951 (0.321)	0.974 (0.339)
		40	80	0.914 (0.244)	0.946 (0.285)	0.981 (0.297)
2.74	0.8	40	30	0.926 (0.199)	0.992 (0.300)	0.919 (0.359)
		40	60	0.944 (0.190)	0.958 (0.248)	0.956 (0.283)
		40	90	0.933 (0.177)	0.971 (0.220)	0.955 (0.245)
3.62	0.9	40	60	0.096 (0.121)	0.994 (0.245)	0.637 (0.324)
		40	80	0.824 (0.123)	0.994 (0.193)	0.837 (0.244)
		40	120	0.910 (0.118)	0.971 (0.169)	0.861 (0.207)
1.08	0.4	60	20	0.882 (0.300)	0.941 (0.360)	0.979 (0.372)
		60	40	0.866 (0.264)	0.932 (0.303)	0.972 (0.309)
		60	60	0.876 (0.247)	0.928 (0.280)	0.969 (0.284)
1.75	0.6	60	20	0.922 (0.259)	0.957 (0.317)	0.978 (0.338)
		60	40	0.921 (0.232)	0.947 (0.267)	0.978 (0.278)
		60	80	0.917 (0.218)	0.945 (0.247)	0.975 (0.255)
2.74	0.8	60	30	0.940 (0.181)	0.980 (0.245)	0.938 (0.283)
		60	60	0.941 (0.167)	0.969 (0.203)	0.959 (0.224)
		60	90	0.935 (0.159)	0.958 (0.188)	0.961 (0.205)
3.62	0.9	60	60	0.807 (0.116)	0.993 (0.193)	0.806 (0.246)
		60	80	0.935 (0.113)	0.987 (0.155)	0.834 (0.187)
		60	120	0.933 (0.109)	0.972 (0.142)	0.900 (0.168)
1.08	0.4	80	20	0.867 (0.250)	0.925 (0.284)	0.967 (0.289)
		80	40	0.867 (0.236)	0.928 (0.266)	0.965 (0.269)
		80	60	0.861 (0.221)	0.921 (0.246)	0.960 (0.248)
1.75	0.6	80	20	0.911 (0.218)	0.942 (0.249)	0.980 (0.259)
		80	40	0.912 (0.208)	0.948 (0.233)	0.974 (0.241)
		80	80	0.908 (0.194)	0.941 (0.216)	0.972 (0.221)
2.74	0.8	80	30	0.939 (0.157)	0.961 (0.189)	0.958 (0.207)
		80	60	0.938 (0.151)	0.965 (0.177)	0.969 (0.191)
		80	90	0.931 (0.143)	0.949 (0.164)	0.963 (0.175)
3.62	0.9	80	60	0.936 (0.105)	0.980 (0.142)	0.884 (0.170)
		80	80	0.942 (0.103)	0.982 (0.133)	0.893 (0.155)
		80	120	0.944 (0.099)	0.968 (0.122)	0.921 (0.140)

We consider the case that the non-diseased group follows a normal distribution with *μ*_*X*_ = 1 and $\sigma_{x}^{2}=1$, and the diseased group follows a gamma distribution with *κ*_*Y*_ = 2, and *θ*_*Y*_ = 0.749, 0.481, 0.259, and 0.153. The *θ*_*Y*_ values are chosen to achieve *J* = 0.4, 0.6, 0.8, and 0.9, respectively. The results are shown in Table 10. The findings are similar to the previous case. Once again, the NP method is preferable due to the satisfactory coverage property.

**Table 10:** Mixed distribution (normal distribution and gamma distribution): coverage probabilities and average widths at the 95% nominal level.

*θ*_*Y*_	*J*	m	n	BAC Coverage (width)	NP Coverage (width)	NPAC Coverage (width)
0.749	0.4	20	20	0.872 (0.355)	0.922 (0.477)	0.995 (0.509)
		20	40	0.859 (0.317)	0.943 (0.416)	0.993 (0.441)
		20	60	0.850 (0.299)	0.944 (0.388)	0.993 (0.413)
0.481	0.6	20	20	0.934 (0.314)	0.974 (0.433)	0.986 (0.479)
		20	40	0.927 (0.274)	0.964 (0.369)	0.981 (0.406)
		20	80	0.930 (0.253)	0.960 (0.341)	0.981 (0.378)
0.259	0.8	20	30	0.869 (0.222)	0.994 (0.353)	0.841 (0.427)
		20	60	0.901 (0.193)	0.990 (0.297)	0.886 (0.356)
		20	90	0.914 (0.175)	0.995 (0.274)	0.894 (0.330)
0.153	0.9	20	60	0.000 (0.135)	0.992 (0.295)	0.603 (0.394)
		20	80	0.036 (0.126)	0.992 (0.247)	0.522 (0.324)
		20	120	0.276 (0.115)	0.996 (0.229)	0.619 (0.301)
0.749	0.4	40	20	0.844 (0.334)	0.923 (0.426)	0.987 (0.447)
		40	40	0.861 (0.289)	0.932 (0.354)	0.983 (0.366)
		40	60	0.839 (0.251)	0.927 (0.303)	0.979 (0.314)
0.481	0.6	40	20	0.919 (0.304)	0.961 (0.389)	0.989 (0.419)
		40	40	0.920 (0.260)	0.951 (0.317)	0.984 (0.336)
		40	80	0.915 (0.219)	0.947 (0.265)	0.981 (0.282)
0.259	0.8	40	30	0.913 (0.219)	0.975 (0.312)	0.933 (0.366)
		40	60	0.941 (0.192)	0.966 (0.249)	0.953 (0.283)
		40	90	0.944 (0.156)	0.977 (0.204)	0.938 (0.233)
0.153	0.9	40	60	0.374 (0.137)	0.989 (0.255)	0.787 (0.331)
		40	80	0.811 (0.130)	0.988 (0.197)	0.812 (0.246)
		40	120	0.871 (0.107)	0.991 (0.162)	0.817 (0.200)
0.749	0.4	60	20	0.844 (0.297)	0.924 (0.360)	0.975 (0.371)
		60	40	0.853 (0.251)	0.931 (0.296)	0.977 (0.303)
		60	60	0.854 (0.228)	0.930 (0.266)	0.972 (0.272)
0.481	0.6	60	20	0.912 (0.271)	0.954 (0.327)	0.985 (0.344)
		60	40	0.919 (0.225)	0.950 (0.263)	0.984 (0.274)
		60	80	0.921 (0.202)	0.952 (0.234)	0.983 (0.244)
0.259	0.8	60	30	0.928 (0.202)	0.972 (0.260)	0.943 (0.293)
		60	60	0.947 (0.167)	0.969 (0.203)	0.965 (0.224)
		60	90	0.952 (0.148)	0.967 (0.179)	0.951 (0.197)
0.153	0.9	60	60	0.833 (0.136)	0.991 (0.205)	0.827 (0.255)
		60	80	0.919 (0.117)	0.989 (0.157)	0.817 (0.188)
		60	120	0.920 (0.102)	0.984 (0.137)	0.875 (0.164)
0.749	0.4	80	20	0.852 (0.242)	0.924 (0.281)	0.967 (0.286)
		80	40	0.854 (0.225)	0.931 (0.259)	0.969 (0.264)
		80	60	0.855 (0.203)	0.927 (0.233)	0.966 (0.237)
0.481	0.6	80	20	0.916 (0.220)	0.949 (0.251)	0.983 (0.260)
		80	40	0.914 (0.202)	0.949 (0.230)	0.977 (0.237)
		80	80	0.910 (0.179)	0.943 (0.203)	0.976 (0.210)
0.259	0.8	80	30	0.942 (0.164)	0.962 (0.194)	0.963 (0.211)
		80	60	0.946 (0.151)	0.960 (0.177)	0.969 (0.191)
		80	90	0.946 (0.132)	0.960 (0.155)	0.960 (0.167)
0.153	0.9	80	60	0.926 (0.115)	0.978 (0.149)	0.875 (0.175)
		80	80	0.940 (0.106)	0.983 (0.134)	0.876 (0.156)
		80	120	0.944 (0.093)	0.985 (0.117)	0.901 (0.136)

The existing methods for confidence intervals all rely on the accuracy of the Youden Index estimate and its variance estimate. The variance can be estimated either from the data or bootstrap samples. The variance of Youden Index estimate is a function of the Youden Index estimate. Therefore, the accuracy of the variance estimate would be significantly affected by the estimate for Youden Index. It has been observed from studies by other researchers [7] that the coverage property is often not satisfied. The Wilson score method is an approach to improve the coverage probability by considering the parameter in the variance as an unknown parameter. The confidence interval from the Wilson score method has to be solved from an inequality. This method has been shown to improve the coverage probability in many statistical problems [15, 11].

### 3.1 An example

We consider an example from a prostate cancer study [16] to compare the three methods for constructing the confidence interval of the Youden Index. It is very important in clinical practice to determine whether the neighboring lymph nodes have been spared or not. The gold standard would be a surgery, which is accurate but very expensive and may be not efficient. In addition, the surgery may cause complications and increase some unnecessary risk for the patient. For this reason, it is of interest to predict the nodal involvement by the level of acid phosphatase in blood serum. 53 total patients were confirmed to have prostate cancer, 20 of them with nodal involvement and the remaining 33 without. For each patient, the level of acid phosphatase in blood serum was measured, and the data can be found in Le [16] as

Patients without nodal involvement (non-diseased group): 40, 40, 46, 47, 48, 48, 49, 49, 50, 50, 50, 50, 50, 52, 52, 55, 55, 56, 59, 62, 62, 63, 65, 66, 71, 75, 76, 78, 83, 95, 98, 102, 187.

Patients with nodal involvement (diseased group): 48, 49, 51, 56, 67, 67,67, 70, 70, 72, 76, 78, 81, 82, 82, 84, 89, 99, 126, 136.

We apply the three methods to the prostate cancer data. The 95% confidence intervals are (0.263, 0.653), (0.253, 0.698), and (0.179, 0.647) for the BAC method, the NP method, and the NPAC method, respectively. The width of the BAC interval is the shortest, followed by the NPAC interval, and the NP interval. As expected, the BAC method has the shortest width since the coverage probability of the BAC method is generally less than the nominal level. The program to conduce this confidence interval calculation is written in R, and is availalbe from the author’s website at: https://faculty.unlv.edu/gshan/.

## Conclusion

In this article, we propose two confidence intervals for the Youden Index using the square-and-add limits based on the Wilson score method, where the parameter in the variance is not estimated as in the existing Wald-type confidence intervals. By conducting extensive Monte Carlo exact simulation studies, we show that the two new intervals generally have better coverage probabilities than the BAC interval. The BAC could perform very poorly for small sample sizes and low *J* values. The performance of the proposed NPAC interval depends on the *J* value, while the NP interval is robust. The coverage of the NP interval is much closer to the nominal level than the other two intervals. In addition, the NP interval is easier to compute than the BAC interval since there is no bootstrap involved. The NP interval is recommended for use in practice.
